# Macrophage derived chemokine (CCL22), thymus and activation-regulated chemokine (CCL17), and CCR4 in idiopathic pulmonary fibrosis

**DOI:** 10.1186/1465-9921-10-80

**Published:** 2009-08-29

**Authors:** Yurika Yogo, Seitaro Fujishima, Takashi Inoue, Fumitake Saito, Takayuki Shiomi, Kazuhiro Yamaguchi, Akitoshi Ishizaka

**Affiliations:** 1 Division of Pulmonary Medicine, Department of Medicine, School of Medicine, Keio University, Tokyo, Japan; 2 Department of Emergency and Critical Care Medicine, School of Medicine, Keio University, Tokyo, Japan; 3 Department of Pathology, School of Medicine, Keio University, Tokyo, JapanSadakazu Aiso, Department of Anatomy, School of Medicine, Keio University, Tokyo, Japan

## Abstract

**Background:**

Idiopathic pulmonary fibrosis (IPF) is a chronically progressive interstitial lung disease of unknown etiology. Previously, we have demonstrated the selective upregulation of the macrophage-derived chemokine CCL22 and the thymus activation-regulated chemokine CCL17 among chemokines, in a rat model of radiation pneumonitis/pulmonary fibrosis and preliminarily observed an increase in bronchoalveolar (BAL) fluid CCL22 levels of IPF patients.

**Methods:**

We examined the expression of CCR4, a specific receptor for CCL22 and CCL17, in bronchoalveolar lavage (BAL) fluid cells, as well as the levels of CCL22 and CCL17, to elucidate their pathophysiological roles in pulmonary fibrosis. We also studied their immunohistochemical localization.

**Results:**

BAL fluid CCL22 and CCL17 levels were significantly higher in patients with IPF than those with collagen vascular diseases and healthy volunteers, and there was a significant correlation between the levels of CCL22 and CCL17 in patients with IPF. CCL22 levels in the BAL fluid did not correlate with the total cell numbers, alveolar lymphocytes, or macrophages in BAL fluid. However, the CCL22 levels significantly correlated with the numbers of CCR4-expressing alveolar macrophages. By immunohistochemical and immunofluorescence analysis, localization of CCL22 and CCR4 to CD68-positive alveolar macrophages as well as that of CCL17 to hyperplastic epithelial cells were shown. Clinically, CCL22 BAL fluid levels inversely correlated with DLco/VA values in IPF patients.

**Conclusion:**

We speculated that locally overexpressed CCL22 may induce lung dysfunction through recruitment and activation of CCR4-positive alveolar macrophages.

## Background

Idiopathic pulmonary fibrosis (IPF), also called usual interstitial pneumonia (UIP) on histological basis, is a chronically progressive interstitial lung disease of unknown etiology, characterized by diffuse interstitial inflammation, fibroblast proliferation with accelerated remodeling of extracellular matrix, and hyperplasia of type II epithelial cells. The prognosis for IPF patients is poor with a median survival of 3-5 years [1-3]. Although several agents such as glucocorticoids, immunosuppressants and pirfenidone, have been administered to IPF patients, less than 30% patients show objective evidence of improvement, and there is no established treatment that certainly improves their outcomes [2-4]. The key pathogenic mechanisms of pulmonary fibrosis are still ill defined, but it is speculated that the disintegration of inflammatory and structural cells, as well as disregulated production of bioactive mediators including cytokines, chemokines, and growth factors, contributes to its pathogenesis [1-3]. Thus, novel therapies based on a novel understanding of its pathophysiology are eagerly awaited.

The thymus and activation-regulated chemokine, CCL17, and the macrophage-derived chemokine CCL22 are members of the CC chemokine family, and CCR4 was identified as their specific receptor [5,6]. CCL17 and CCL22 have been recognized as Th2 chemokines, and their involvement in allergic diseases, such as atopic dermatitis, bronchial asthma and eosinophilic pneumonia has been revealed [7,8]. However, there is increasing evidence that these two chemokines are also involved in the pathophysiology of pulmonary fibrosis. Belperio et al. demonstrated that CCL17, CCL22 and CCR4 were overexpressed in a mice model of bleomycin-induced pulmonary fibrosis [9], and Pignatti et al. showed that CCR4 expression on bronchoalveolar lavage (BAL) fluid CD4 T cells were significantly elevated in IPF patients [10]. We have previously demonstrated the selective upregulation of CCL22 and CCL17 in a rat model of radiation pneumonitis/pulmonary fibrosis [11]. In this model, CCL22 and CCL17 were localized primarily to alveolar macrophages, whereas CCR4 was expressed by alveolar macrophages as well as lymphocytes. In addition, we observed elevated levels of CCL22 in BAL fluid of IPF patients by preliminary experiments. Thus, the current study was aimed to further elucidate the role of CCL22 and CCL17 in IPF. We determined CCL22 and CCL17 levels in BAL fluid using new sensitive ELISAs, and analyzed their correlation with clinical parameters. Furthermore, we analyzed CCR4 expression on BAL fluid cells and obtained supportive results that CCL22 and CCR4 contribute to the pathophysiology of IPF.

## Materials and methods

### Study Population

We studied 19 patients with IPF (18 males and 1 female, mean age 67.0 ± 1.9 years, SEM), 6 with sarcoidosis (3 males and 3 females, mean age 58.5 ± 23.2 years), and 9 with collagen vascular diseases associated with interstitial pneumonia (CVD-IP; 3 males and 6 females, mean age 59.4 ± 14.8 years), along with 6 non-smoking healthy volunteers without any medication in the previous six months (6 males, aged between 20 and 24 years). After obtaining informed consent from all patients and healthy volunteers, BAL was performed by a standard procedure. BAL total cell numbers were counted and differential cell counts were analyzed. The study was approved by the Ethical Committee of the School of Medicine, Keio University.

IPF was diagnosed, according to the diagnostic criteria by American Thoracic Society (ATS)/European Respiratory Society (ERS), for cases that satisfied all four major criteria: (1) exclusion of other known causes of interstitial lung disease; (2) abnormal pulmonary function; (3) bibasilar reticular abnormalities with minimal ground-glass opacities on high resolution computed tomography (HRCT) scans; (4) transbronchial lung biopsy specimen or BAL fluid showing no features to support an alternative diagnosis [3]. In addition, at least three of the four minor criteria had to be fulfilled: (1) age>50 years; (2) insidious onset of otherwise unexplained dyspnea on exertion; (3) duration of illness >3 months; (4) bibasilar, inspiratory crackles. Open lung biopsy was performed in one IPF patient, and transbronchial lung biopsy (TBLB) in 11 patients without any atypical findings. No patients showed any atypical findings in BAL fluid cell analysis, nor symptoms or signs of respiratory tract infection, and none had been treated with corticosteroids or immunosuppressants. We excluded patients who showed massive lung honeycombing on chest X-rays or chest CT scans, and those with an acutely exacerbating clinical course.

Sarcoidosis was diagnosed from chest X-ray findings, BAL fluid differential cell counts, and histological findings from TBLB. Non-caseous granulomas were confirmed by TBLB in all patients.

CVD-IP was diagnosed according to the criteria of the American College of Rheumatology. Two patients with rheumatoid arthritis (RA), 1 with polymyositis (PM)/dermatomyositis (DM), 2 with mixed connective tissue disease (MCTD), 2 with systemic sclerosis (SSc), and 2 with Sjogren's syndrome (SjS) were included in the study.

### Lung Function Tests and Lung Fibrosis Scores on Chest X-Rays

Spirometry was performed for all IPF and sarcoidosis patients and 8 patients with CVD-IP. Single-breath carbon monoxide diffusing capacity (DLco) was evaluated in 15 patients with IPF, 5 with sarcoidosis, and 5 with CVD-IP. PaO_2_, PaCO_2_, and alveolar-arterial oxygen gradient (AaDO_2_) were evaluated in 16 patients with IPF. In addition, scores for pulmonary fibrosis were assigned from chest X-rays following a previously described method [12].

### BAL Fluid CCL22 and CCL17 Analysis

CCL22 and CCL17 concentrations in BAL fluids were determined by sensitive sandwich ELISAs according to the manufacturer's protocols (GT Development Co., Seattle WA). The absorbance at 450 nm was determined on a microplate reader (SPECTRAFluor Plus, Tecan Co., Minneapolis, MN), and the concentrations were determined by interpolation of their absorbance from the standard curve. Each sample was tested in triplicate and the mean value was obtained. The detection limit for both CCL22 and CCL17 was 6.3 pg/ml.

### Flow Cytometric Analysis of BAL Fluid Cell Subpopulations

For flow cytometric analysis, 5 × 10^5 ^BAL cells were suspended in 100 μl phosphate-buffered saline (PBS) and incubated with (FITC)-conjugated anti-human CD4 monoclonal antibody (cat. #551120, Becton, Dickinson, Franklin Lakes, NJ) and phycoerythrin-conjugated anti-human CCR4 monoclonal antibody (Becton, Dickinson) for 30 min. After incubation, the cells were washed twice with PBS, and analyzed using a flow cytometer following the previously established protocol (Epics XL•MC L, Beckman Coulter, Inc., Fullerton, CA) [13,14]. Alveolar macrophages were primarily identified on a forward and side scattergram, and we additionally used CD4 as a marker of alveolar macrophages as well as helper T lymphocytes to better eliminate contaminated neutrophils and debris. A weakly CD4-positive cell population was gated [15], and the expression of CCR4 was analyzed.

### Histological and Immunohistochemical Examination

For histological and immunohistochemical analysis, we used lung tissue obtained through TBLB or open lung biopsy. The lung tissue was fixed with 10% formalin, embedded in paraffin, and the paraffin sections were stained with hematoxylin and eosin (HE). For immunohistochemistry, the sections were stained with specific goat polyclonal antibodies against human CCL22, CCL17 (Santa Cruz Biotechnology Inc, Santa Cruz, CA), CCR4 (Abcam, Cambridge, UK), or monoclonal antibody for human CD68 (KP1, Santa Cruz Biotechnology Inc) [16,17], using an indirect streptavidin-biotinylated complex method. We additionally performed immunofluorescence staining using Alexa-488- and Cy3-labeld secondary antibody to show the colocalization of CCL22, CCR4 and CD68. In these analyses, DAPI was used for the staining of nuclei.

### Statistical Analysis

All data are presented as mean ± SEM. A one-way analysis of variance (ANOVA) followed by Fisher's least significant difference (LSD) test was applied to detect statistically significant differences among groups. Significant differences were accepted at p < 0.05.

## Results

### Patient Characteristics and BAL Fluid Analysis

Clinical characteristics as well as BAL fluid data of the patients are summarized in Tables 1 and 2. DLco/VA was significantly lower in patients with IPF than in those with CVD-IP. The total BAL fluid cell number was significantly higher in patients with CVD-IP than in the other groups. The percentage of BAL fluid macrophages was significantly lower in IPF, CVD-IP and sarcoidosis patients than in healthy volunteers, and it was significantly lower in CVD-IP patients than in IPF patients. Patients with sarcoidosis and CVD-IP showed a significantly increased percentage of BAL fluid lymphocytes than those with IPF and healthy volunteers. The percentage of BAL fluid neutrophils was significantly higher in patients with CVD-IP than in the other groups. The percentage of BAL fluid eosinophils was significantly higher in patients with IPF than those with sarcoidosis and healthy volunteers.

**Table 1:** Patient Characteristics and Lung Functions

	IPF	Sar	CVD-IP	HV
Male/female	18/1	3/3	3/6	6/0

Age (range)	67.0 ± 1.9 (48-83)	58.5 ± 23.2 (24-76)	59.4 ± 14.8 (33-76)	N.D. (20-24)

Smoker	16*^†^	6*	3	0

PaO_2_/FIO_2_	372 ± 9.2 (307-453)	419 ± 29 (319-529)	358 ± 23 (278-448)	N.D.

%VC	62 ± 4.6 (33-110)	101 ± 6.1^#^ (83-120)	67 ± 6.6 (43-98)	N.D.

DLCO/VA	4.0 ± 0.2^†^ (2.6-5.4)	4.8 ± 0.4 (4.1-6.3)	7.1 ± 1.9 (4.4-14.0)	N.D.

IPF, idiopathic pulmonary fibrosis; Sar, sarcoidosis; CVD-IP, collagen vascular disease with interstitial pneumonia; HV, healthy volunteers; N.D, not determined; DLco, single-breath carbon monoxide diffusing capacity; VA, alveolar ventilation per minute

Age data and lung function parameters are shown as mean ± SEM.

*p < 0.001 *v. s*. HV

^§^p < 0.001 *v. s*. CVD-IP, ^†^p < 0.005 *v. s*. CVD-IP

^#^p < 0.0005 *vs. *IPF

**Table 2:** BAL Fluid Cell Characteristics

	IPF (n = 19)	Sar (n = 6)	CVD-IP (n = 8)	HV (n = 6)
Total cells (10^5^/ml)	6.2 ± 0.8 (1.9-14.8)	4.9 ± 0.3 (4.0-6.0)	11.2 ± 3.1*^#£^ (1.1-27.9)	2.7 ± 0.5 (0.6-4.0)

Macrophage (%)	78.0 ± 2.6^∫^ (60.2-97.0)	62.9 ± 10.8* (29.5-95.0)	44.0 ± 9.9^"§ ^(5.5-74.5)	95.6 ± 0.3^§$^ (94.7-96.6)

Lymphocyte (%)	11.3 ± 2.1 (0-27.4)	34.6 ± 10.5*^‡^ (5.0-68.5)	33.8 ± 8.7*^‡^ (12.0-87.5)	3.1 ± 0.2 (2.6-4.0)

Neutrophil (%)	6.1 ± 1.4 (1.0-23.0)	1.7 ± 0.8 (0-4.0)	18.4 ± 8.6^|#£^ (0-65.5)	1.1 ± 0.1 (0.7-1.6)

Eosinophil (%)	4.4 ± 1.1^∫#^ (0-14.5)	0.5 ± 0.3 (0-1.9)	1.7 ± 0.9 (0-7.5)	0.2 ± 0.2 (0-0.9)

CD4/CD8	3.1 ± 0.6 (0.2-9.6)	11.2 ± 4.0^&¥^ (2.4-29.3)	1.8 ± 0.5 (0.4-3.9)	N.D.

IPF, idiopathic pulmonary fibrosis; Sar, sarcoidosis; CVD-IP, collagen vascular disease with interstitial pneumonia; HV, healthy volunteers; N.D., not determined

All data were shown as mean ± SEM.

"p < 0.0001 *v.s*. HV, *p < 0.005 *v.s*. HV, ^∫^p < 0.05 *v.s*. HV

^†^p < 0.0005 *v.s*. CVD-IP, ^&^p < 0.005 *v.s*. CVD-IP

^$^p < 0.005 *v.s*. Sar, ^#^p < 0.05 *v.s*. Sar

^§^p < 0.0001 *v.s*. IPF, ^¥^p < 0.001 *v.s*. IPF, ^‡^p < 0.005 *v.s*. IPF, ^£^p < 0.05 *v.s*. IPF

### BAL Fluid Chemokines, Cell Differentials and Subpopulations

**Figure 1:**
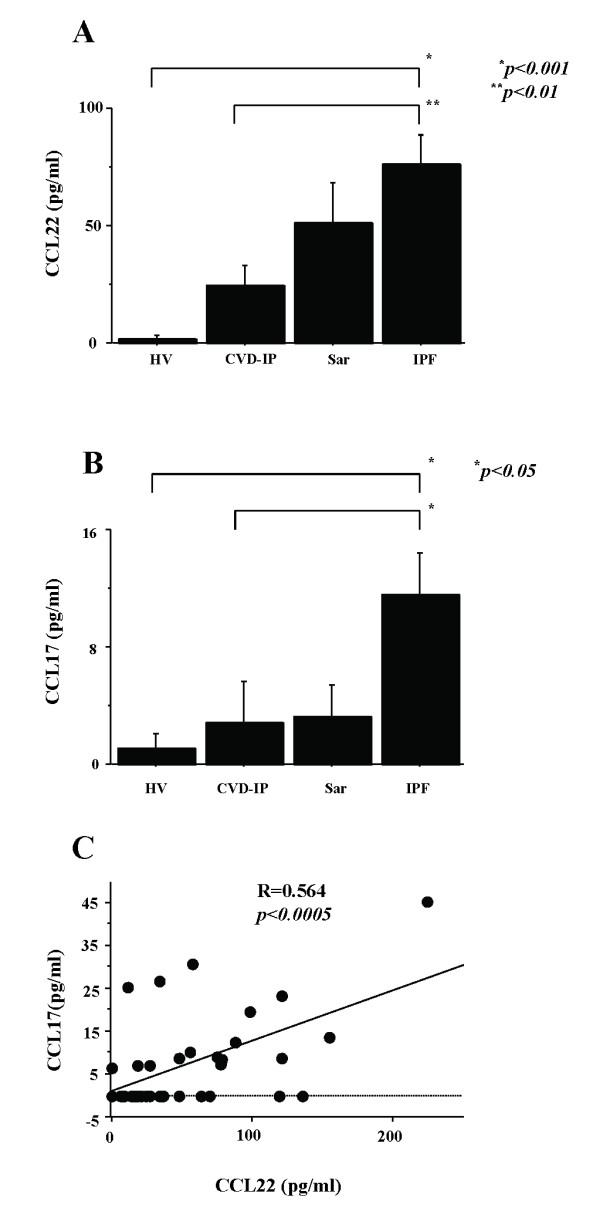
**BAL fluid CCL22 and CCL17 in fibrotic lung diseases**. BAL fluid levels of CCL22 and CCL17 were determined by sensitive ELISAs. CCL22 and CCL17 levels were significantly higher in patients with idiopathic pulmonary fibrosis (IPF) than in those with CVD-IP and healthy volunteers (A, B). In IPF patients, BAL fluid CCL22 levels correlated significantly with CCL17 levels (C). IPF, idiopathic pulmonary fibrosis; HV, healthy volunteers; CVD-IP, collagen vascular disease with interstitial pneumonia; Sar, sarcoidosis.

CCL22 and CCL17 BAL fluid levels were significantly higher in patients with IPF than in those with CVD-IP and healthy volunteers (Fig 1A, B). CCL22 BAL fluid levels were significantly correlated with CCL17 levels in IPF patients (Fig 1C). We found no correlation of CCL22 and CCL17 with the total cell numbers and differential cell counts in BAL fluid.

**Figure 2:**
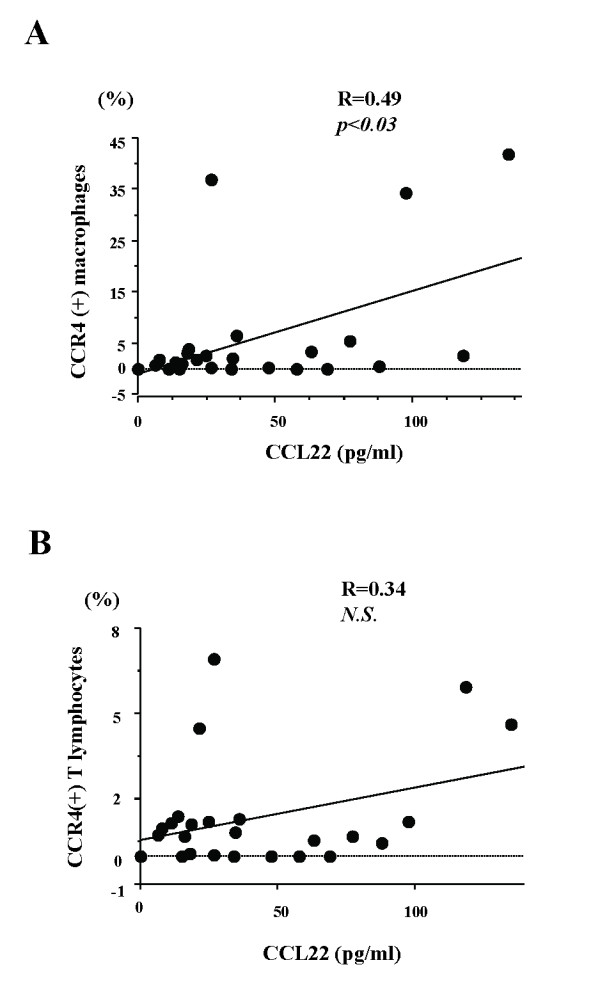
**Correlations between BAL fluid CCL22 and CCR4-positive alveolar macrophages and lymphocytes in all patients examined**. To further elucidate the roles of the chemokines in recruiting cells to the lungs in fibrotic lung diseases, we analyzed CCR4-positive BAL fluid cell subpopulations by flow cytometry in IPF. CCL22 levels significantly correlated with the number of CCR4-positive alveolar macrophages (A). CCL22 levels in IPF patients were significantly correlated with the number of CCR4-positive alveolar macrophages and lymphocytes. These correlations were not observed between these subpopulations and BAL fluid CCL17 levels.

To further elucidate the roles of these chemokines in recruiting cells to the lungs in fibrotic lung diseases, we analyzed CCR4-positive BAL fluid cell subpopulations by flow cytometry. CCL22 levels were significantly correlated with the total number of CCR4-positive BAL fluid cells in all patients examined. Furthermore, CCL22 levels were significantly correlated with the number of CCR4-positive alveolar macrophages (Fig 2A), but not with lymphocytes (Fig 2B). These correlations were not observed between these subpopulations and CCL17 BAL fluid levels. CCL22 levels in IPF patients were significantly correlated with the number of CCR4-positive alveolar macrophages (R = 0.87, p < 0.001) and CCR4-positive lymphocytes (R = 0.75, p < 0.01). In contrast, BAL fluid CCL17 levels did not correlate with CCR4-positive alveolar macrophages or lymphocytes in IPF patients.

### Immunohistochemical Localization of CCL22, CCL17, and CCR4 in IPF

**Figure 3:**
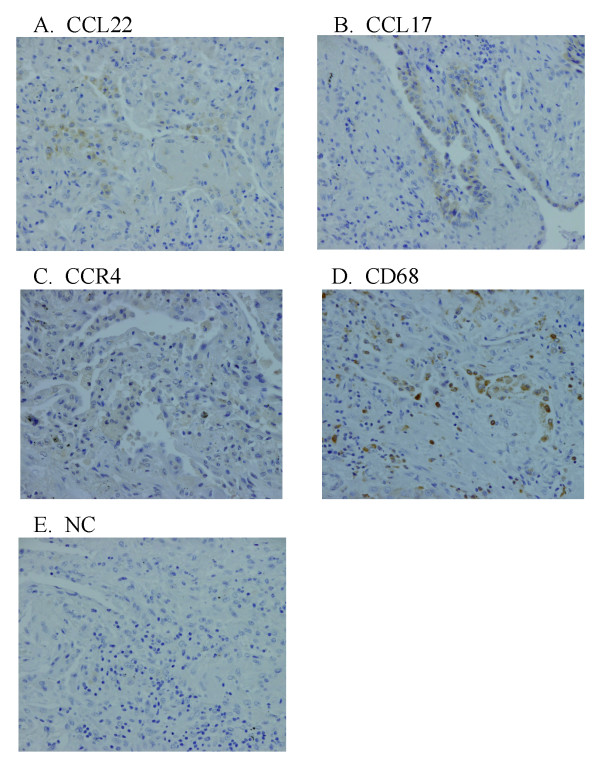
**Lung immunohistochemical photomicrograph of CCL17, CCL22, CCR4, and CD68 in patients with idiopathic pulmonary fibrosis (IPF)**. We examined the localization of CCL17, CCL22, CCR4, and CD68 by immunohistochemistry. The sections were initially incubated with anti-CCL22 antibody (A), anti-CCL17 antibody (B), anti-CCR4 antibody (C), anti-CD68 antibody (D), or their diluent buffer (E), and then stained using an indirect streptavidin-biotinylated complex method. A fraction of the alveolar macrophages was positive for CCL22, whereas CCL17 was exclusively expressed by some hyperplastic epithelial cells (A, B). There were few alveolar macrophages which were weakly positive for CCR4 (C). The tissue distribution of alveolar macrophages was confirmed by their positivity for CD68 (D). In contrast, no lung cells were positively stained in negative control (NC) sections (E).

We also examined the localization of CCL22, CC17, and CCR4 by immunohistochemistry. A fraction of alveolar macrophages were positive for CCL22, whereas CCL17 was exclusively expressed by hyperplastic epithelial cells (Fig 3A, B). CCR4 also seemed to be weakly positive for a part of alveolar macrophages (Fig 3C). CD68, a specific marker of macrophages, was localized in the cells identical or similar to CCL22- and CCR4-positive cells (Fig 3D). There were very few lymphocytes, and CCR4-positive lymphocytes were barely found.

**Figure 4:**
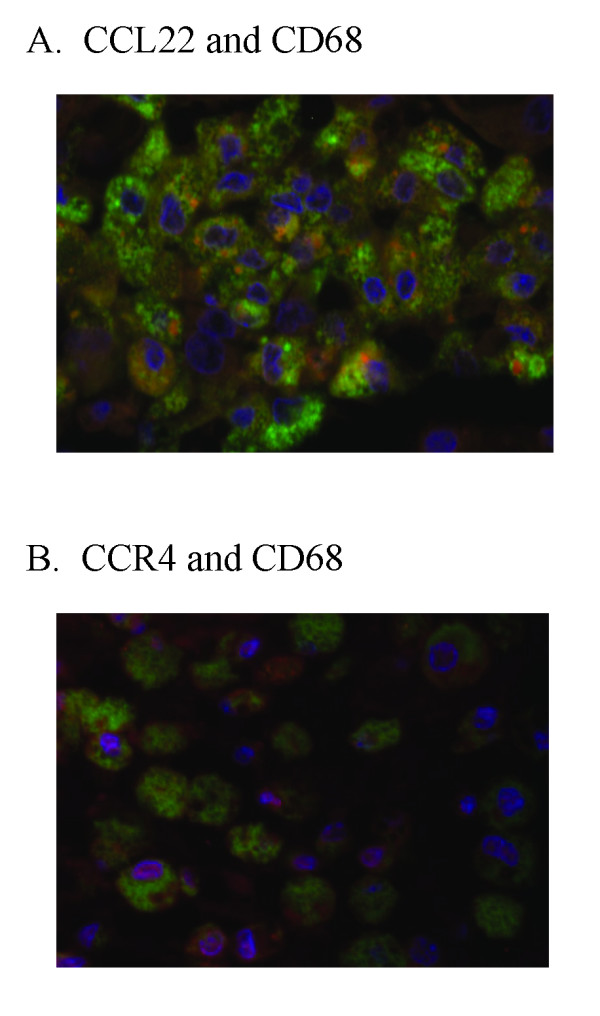
**Lung immunofluorescence photomicrograph of CCL22 and CCR4 in patients with idiopathic pulmonary fibrosis (IPF)**. We examined the localization of CCL22 and CCR4 in CD68-positive alveolar macrophages by a dual immunofluorescence technique. A. Localization of CCL22 (red) to a certain fraction of CD68 (green) -positive alveolar macrophages was shown. B. Localization of CCR4 (red) to a small fraction of CD68 (green) -positive alveolar macrophages was shown. Nuclei were counterstained with DAPI (blue).

To further confirm the localization of CCL22 and CCR4 to alveolar macrophages, we used dual immunofluorescence staining technique. Localization of CCL22 and CCR4 to a fraction of CD68-positive alveolar macrophages was shown (Fig 4A, B). These observations suggested that alveolar macrophage-derived CCL22 as well as epithelial cell-derived CCL17 contribute to the recruitment and activation of CCR4-positive cells, which are probably alveolar macrophages in IPF patients.

### Correlation between BAL Fluid Chemokines and Clinical Parameters

We further examined the correlation between the BAL fluid chemokines and various clinical parameters, including serum lactate dehydrogenase, C-reactive protein, KL-6, and semi-quantitative scores of chest X-ray abnormalities in IPF patients. We assessed the degree of radiographic abnormalities according to Watter's method [12]. Briefly, areas of abnormal shadows, presence of honeycombing, and the diameter of the main pulmonary artery were assessed by expert pulmonologists, and a semi-quantitative radiological score was calculated for each patient. However, we did not find any significant correlations between any of the clinical parameters examined and the CCL22 and CCL17 levels in BAL fluid.

**Figure 5:**
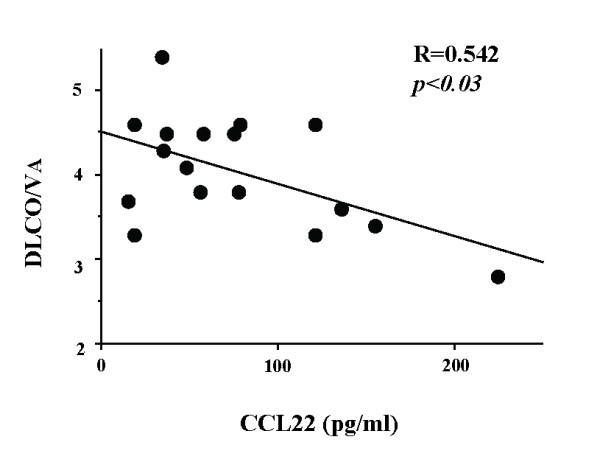
**Correlation between BAL fluid CCL22 and lung diffusing capacity in idiopathic pulmonary fibrosis (IPF) patients**. We examined the correlation of BAL fluid chemokines with indices of lung function tests in IPF patients. An inverse correlation was observed between BAL fluid CCL22 levels and DLco/VA values. Although BAL fluid CCL17 also tended to correlate inversely with DLco/VA, there was no statistical significance. DLco, single-breath carbon monoxide diffusing capacity; VA, alveolar ventilation per minute.

We next examined the correlation of the BAL fluid chemokines with indices of lung function tests in IPF patients. An inverse correlation was observed between BAL fluid CCL22 levels and DLco/VA values (Fig 5). Although BAL fluid CCL17 also tended to correlate inversely with DLco/VA, no statistical significance was present. There were no significant correlations between the two BAL chemokines levels and other parameters of lung function, including %VC and PaO_2_/FIO_2_.

## Discussion

In the present study, we examined the T-helper 2 (Th2) chemokines, CCL22, CCL17, and BAL fluid cells expressing CCR4, a specific receptor for these chemokines, to elucidate their pathophysiological roles in IPF patients. We also studied the localization of CCL22, CCL17, and CCR4 by immunohistochemistry. The levels of CCL22 and CCL17 in BAL fluid were significantly higher in patients with IPF than in those with CVD-IP and healthy volunteers, and there was a significant correlation between the levels of CCL22 and CCL17 in IPF. CCL22 levels in the BAL fluid did not correlated with total cell numbers, alveolar lymphocytes, and macrophages in the BAL fluid. However, the CCL22 levels were significantly correlated with the numbers of CCR4-expressing alveolar macrophages. By immunohistochemical analysis, localization of CCL22 and CCR4 to alveolar macrophages as well as that of CCL17 to hyperplastic epithelial cells were shown. Clinically, CCL22 levels in BAL fluid inversely correlated with DLco/VA values in IPF patients. Collectively, we speculated that locally overexpressed CCL22 may contribute to the induction of lung dysfunction mainly through recruitment of CCR4-positive alveolar macrophages.

### Increased Production of CCL17and CCL22 in IPF

In our previous study, we showed that the production of CCL22 and CCL17 in rat radiation pneumonitis increased significantly, but CCL17 was undetectable in BAL fluid of IPF patients [11]. Previous reports found no significant increase in BAL fluid CCL17 [9,18]. Using a more sensitive ELISA kit in the current experiment, we confirmed significant increases in CCL17 and CCL22 BAL fluid levels in IPF patients as compared with those in CVD-IP patients and healthy volunteers. The levels of CCL17 were lower than those of CCL22 in 14 out of 16 patients examined, and there was a significant correlation between the two levels, suggesting a common stimulus or stimuli for their induction.

In our study, CCL17 was positive in hyperplastic epithelial cells. Our results regarding CCL17 were consistent with previous observations in IPF [9,19], and CCL17 detected in BAL fluid could be mainly derived from these cells. Bronchial epithelial cells are the major source of CCL17 under physiological and pathological conditions, including bronchial asthma [20], and CCL17 is inducible by various stimuli, such as TNF-alpha, interleukin (IL)-4, interferon-gamma, and TGF-beta [21,22]. Because overproduction of these cytokine has been shown previously, they also could be *in vivo *stimuli for CCL17 in IPF.

Our study revealed that immunoreactive CCL22 was predominantly localized to alveolar macrophages, whereas Marchal-Sommé et al reported that CCL22 was positive in hyperplastic epithelial cells, fibroblasts, and endothelial cells, but not in alveolar macrophages [19]. However, because our previous study showed the localization of CCL22 to alveolar macrophages in a rat radiation pneumonitis model [11], and the augmented production of CCL22 was shown in IPF [23], it is reasonable to speculate that alveolar macrophages are at least partly responsible for high levels of CCL22 in IPF. CCL22 is inducible in alveolar macrophages by IL-4, PGE_2_, and TGF-beta [24]. Because overproduction of these mediators has been shown previously [25], they may be *in vivo *inducers of CCL22 in IPF.

### Possible Contribution of Lung CCL22 to the Recruitment of CCR4-Positive Alveolar Macrophages

In the present study, we found that BAL fluid levels of CCL22 were significantly correlated with the number of CCR4-positive alveolar macrophages among all patients examined. CCL22 levels in IPF patients were significantly correlated with the number of CCR4-positive alveolar macrophages and lymphocytes. Thus, although the percentage of CCR4-positive cells was relatively small among alveolar macrophages, the results may indicate that locally overproduced CCL22, but not CCL17, contributes to the recruitment of alveolar macrophages, and to a lesser extent, alveolar lymphocytes to the lungs in IPF patients.

In animal models of pulmonary fibrosis, we have found CCR4 expressed on alveolar macrophages in rat radiation pneumonitis/pulmonary fibrosis, and Belperio et al. demonstrated predominant CCR4 expression on alveolar macrophages in mice bleomycin-induced pulmonary fibrosis [9]. Furthermore, Trujillo et al. recently demonstrated that bleomycin induced CCL17-dependent activation of CCR4 in alveolar macrophages using CCR4-deficient mice [26]. Thus, the CCL22-CCR4 axis may contribute to the activation of alveolar macrophages in pneumonitis and pulmonary fibrosis.

### Inverse Correlation of BAL Fluid CCL22 with Lung Diffusing Capacity in IPF

Our current study demonstrated that CCL22 was inversely correlated with DLco/VA. Because DLco/VA is affected by both total surface area and thickness of alveolar walls, and these regions are the major targets of alveolar macrophage infiltration in IPF, the results may suggest that alveolar macrophage recruitment by CCL22 induces a dose-dependent decrease in DLco/VA. It is also possible that CCL22 or CCR4-positive alveolar macrophages are involved in the destruction of lung parenchyma in IPF.

Previously, Pignatti et al. demonstrated an increase in CCR4-positive alveolar T-lymphocytes and their inverse correlation with DLco in IPF [10]. In contrast, the increase of CCR4 expression on T-lymphocytes was relatively small and we did not find their significant correlation with the parameters of lung functions, including DLco in our study. The discrepancy between their and our results may be derived from the difference in disease stages or characteristics. All of our patients were in a stable stage, and we excluded the patients who showed massive lung honeycombing, or were treated with corticosteroids, whereas they did not exclude such patients. In addition, the CCR4-expressing alveolar macrophages, as well as BAL fluid CCL22 levels, were not examined in their study. Since we also found a significant correlation between BAL fluid CCL22 levels and CCR4-positive lymphocytes in IPF patients, it is possible to speculate that locally overproduced CCL22 contributes to the recruitment of CCR4-positive alveolar macrophages, and to a lesser extent, to the recruitment of CCR4-positive alveolar T-lymphocytes.

## Conclusion

CCL22 and CCL17 were both increased in BAL fluid of IPF patients and CCL22 levels in BAL fluid correlated proportionally with the numbers of CCR4-positive alveolar macrophages, and inversely with DLco/VA. CCL22 may contribute to the recruitment and activation of alveolar macrophages, and consequently to the destruction of lungs in patients with IPF.

## List of Abbreviations

AaDO_2_: alveolar-arterial oxygen gradient; BAL: bronchoalveolar lavage; CVD-IP: collagen vascular disease with interstitial pneumonia; ELISAs: enzyme-linked immunosorbent assay; DLco: single-breath carbon monoxide diffusing capacity; HV: healthy volunteers; IPF: idiopathic pulmonary fibrosis: N.D: not determined; Sar: sarcoidosis; TBLB: transbronchial lung biopsy; UIP: usual interstitial pneumonia; VA: alveolar ventilation per minute

## Competing interests

The authors declare that they have no competing interests.

## Authors' contributions

YY primarily collected and analyzed the data, with the help of TI and FS. This manuscript was prepared by YY under SF's instruction. TS was involved in pathological diagnosis and immunohistochemical analysis. SA contributed to FACS analysis and interpretation of data. This study was supported by the scientific fund for KY, AI, and SF.
